# MQL Strategies Applied in Ti-6Al-4V Alloy Milling—Comparative Analysis between Experimental Design and Artificial Neural Networks

**DOI:** 10.3390/ma13173828

**Published:** 2020-08-30

**Authors:** Nelson Wilson Paschoalinoto, Gilmar Ferreira Batalha, Ed Claudio Bordinassi, Jorge Antonio Giles Ferrer, Aderval Ferreira de Lima Filho, Gleicy de L. X. Ribeiro, Cristiano Cardoso

**Affiliations:** 1 Faculty of Mechatronic Technology, National Service for Industrial Training (SENAI-SP), São Caetano do Sul, SP 09572-300, Brazil; jorge.ferrer@sp.senai.br (J.A.G.F.); aderval.filho@sp.senai.br (A.F.d.L.F.); 2 Department of Mechatronics and Mechanical Systems Engineering, Polytechnic School of Engineering of the University of Sao Paulo (USP), São Paulo, SP 05508-900, Brazil; gilmar.batalha@usp.br; 3 Department of Mechanical Engineering, University Center of the Mauá Institute of Technology (IMT), São Caetano do Sul, SP 09580-900, Brazil; 4 Technology Faculty of Mauá, The Paula Souza State Center for Technological Education (CEETEPS), São Paulo, SP 09390-120, Brazil; 5 Department of Mechanical Engineering, University Center of Educational Foundation of Ignatius (FEI), São Bernardo do Campo, SP 09850-901, Brazil; 6 Department of Mechanical Engineering, University of Taubaté (UNITAU), Taubaté, SP 12020-270, Brazil; 7 Institute for Innovation in Advanced Manufacturing and Microfabrication, National Service for Industrial Training (SENAI-SP), São Paulo, SP 04757-000, Brazil; gleicy.ribeiro@sp.senai.br (G.d.L.X.R.); cristiano.cardoso@sp.senai.br (C.C.)

**Keywords:** Ti-6AL-4V, MQL, machining, milling, lubrication, optimization

## Abstract

This paper presents a study of the Ti-6Al-4V alloy milling under different lubrication conditions, using the minimum quantity lubrication approach. The chosen material is widely used in the industry due to its properties, although they present difficulties in terms of their machinability. A minimum quantity lubrication (MQL) prototype valve was built for this purpose, and machining followed a previously defined experimental design with three lubrication strategies. Speed, feed rate, and the depth of cut were considered as independent variables. As design-dependent variables, cutting forces, torque, and roughness were considered. The desirability optimization function was used in order to obtain the best input data indications, in order to minimize cutting and roughness efforts. Supervised artificial neural networks of the multilayer perceptron type were created and tested, and their responses were compared statistically to the results of the factorial design. It was noted that the variables that most influenced the machining-dependent variables were the feed rate and the depth of cut. A lower roughness value was achieved with MQL only with the use of cutting fluid with graphite. Statistical analysis demonstrated that artificial neural network and the experimental design predict similar results.

## 1. Introduction

Machining processes, such as turning, boring, drilling, and milling, are some of the most important techniques used in industries [1]. In these processes, the material is separated from the original body by the chips, creating a new shape, and it is estimated that it contributes to approximately 5% of the GDP in developed countries [2]. This removal of wanted parts leads to plastic deformations of work specimens, and most of the fed energy is converted into heat [3].

Considering the importance of these processes in the industry, mainly in aerospace, which is highly specialized, it is necessary to keep resources and developments to meet the demand when it arises [4]. Most materials used in the aerospace industry are hard to machine [5]. The Ti-6Al-4V alloy with an α-β microstructure is estimated to account for 50% of the global titanium metal production, and 80% of this corresponds to the aerospace and medical industries [6,7]. According to [8], 14% of the aerostructure of the Airbus A350-900 XWB [2], 15% of the Boeing 787 [3], and 25% of the GE CF6 aeroengine is made from titanium alloys. In addition to the aerospace industry, titanium is also used in the marine, biomedicine, energy, automobile, and chemical industries [9,10]. The main properties of titanium are a high hot strength and hardness, and a superior corrosion resistance and fracture toughness when compared with other materials. It has the highest strength-to-weight ratio amongst all structural materials, excellent creep and biocompatibility, a low elastic modulus, and chemical inertness at room temperature [11]. As a result of these properties, titanium is considered a hard-to-cut material [12], and the main problems include the fact that the high chemical reactivity makes chips easily adhere to the tool-cutting edge, and the low thermal conductivity (1/6 of that for steel) increases the temperature at the tool’s cutting edge (which can easily reach beyond 1000 °C) [13,14,15,16]. A low modulus of elasticity and an extreme strength at high temperature generate long ductile chips, a relatively large contact length between the chip and the cutting tool, and a high compressive stress, leading to a poor tool life, or to higher cutting forces [17].

Since heat generated during machining is a major problem, it is important to choose an adequate lubrication/cooling condition, to improve the machinability of a difficult-to-cut material [12,18]. The minimum quantity lubrication (MQL) is an attractive alternative solution for floods, which have occurred for decades and bring health risks to workers [17,19], and for dry machining, which can accelerate tool wear and the degradation of piece surface integrity [11]. This technique has also been used more often, due to pressure from governments, law enforcement, and environmental agencies that have compelled manufacturers to adopt it [20]. It uses a small amount of cutting fluid in the form of an oil mist that is directly sprayed to the contact zone, helping to reduce friction and cutting forces, and decreasing the surface’s crumple zone by a large amount (~50%) when compared with dry cutting [21]. Consequently, MQL reduces tool wear [22,23]. This technique combines the lubrication from the cutting fluid and the cooling from the air pressure, sometimes reducing the thermal shock caused by flood lubrication [24], and can be used in drilling, milling, turning, and others. Studies conducted by [25] also showed a minimization in vibrations along with radial force during turning.

Artificial neural networks (ANNs) model the complex nonlinear relationships between input and output parameters by observing datasets and identifying patterns, without the need to write explicit programs [4]. An ANN is inspired by the way biological nerves, such as the brain, work to solve problems, and the first artificial neuron was produced in 1943 by McCulloch and the logician Walter Pits [26,27]. The ANN and the genetic algorithm (GA) are important alternatives to be used in machining processes, due to their high complexity in optimizing cutting parameters. They have become very popular in recent years due to their capability of learning nonlinear behavior [28], with many studies conducted. The authors in [27] studied ANNs to predict the material removal rate and surface roughness in the CNC milling of P20 steel and the results indicating a successful application. The authors in [9] used a backpropagation neural network (BPNN), and optimized the overall cutting performance during the high-speed turning of the Ti-6Al-4V alloy, and the results achieved a balance among all studied responses. The authors in [18] investigated the characteristics of different nano-cutting fluids, molybdenum disulfide, and graphite under MQL conditions, using Box–Cox transformation, normal probability plots, and analysis of variance (ANOVA) tests. The best choice for the application was chosen, and the machining characteristics of the Inconel-800 alloy were improved. The authors in [17] studied turning tests with a combination of cooling techniques on titanium (Grade 2). The surface quality measurements, force values, and tool wear were investigated using a combination of ANOVA, and the correlations were established with a success rate of 90%. The authors in [28] used regression analysis and an ANN to predict the surface roughness during the hard turning of AISI 52100, and found superior results in the ANN application. The authors in [4] used the friction force computed for 648 experimental trials to develop and optimize ANN architecture prediction during turning with different materials and cutting parameters, and obtained a 12.5% difference between the estimated and measured values. The results obtained in [24] were even better, with variations from 1% to 4% during a comparative experimental study in three different machining environments (dry air cooling, flood cooling, and minimum quantity cutting fluid). The authors in [29] studied the dry machining of hardened steel En31 to identify the influence of input parameters (speed, feed rate, and the depth of cut) on the surface roughness, and it was possible to analyze the process sensitivity. The authors in [30] used an ANN to predict the cutting force by monitoring the spindle and the individual drives, obtaining success in application during 2.5D milling in E355 steel. The authors in [31] also used an ANN and a GA to predict the minimum surface roughness and chose the best cutting parameters to achieve it. The authors in [32] described numerous papers in which an ANN was developed using this modern technique for many applications besides machining, showing its versatility.

Over time, statistical tools have been applied and have helped to assist in the process of analyzing problems and making decisions. The design of experiments (DOE) has often been used to design a specific experiment or to define data in a methodology. The collection and organization of data for the experiment should ensure that the process has the best possible yield and that there is safety or a minimal difference in the empirical results related to the collected data.

As demonstrated by a growing number of articles published in the last decade, many researchers in the manufacturing field explore ways of applying artificial neural networks to control or to perform an estimation of a product’s critical quality, and to optimize production processes [33]. In this respect, many authors use DOE techniques to implement and optimize ANN parameters. According to authors in [34] the combination of multilayer perceptron with DOE has proven to be an appropriate tool in modeling and problem analysis. Chromium layer thickness predictions on hard chrome plating processes´ results suggest that DOE may be successfully used for the optimization of ANNs´ backpropagation parameters. In another paper on turning, DOE was employed to determine factor levels that benefit network forecasting skills, concluding that the DOE methodology constitutes a better approach to the design of RBF networks for roughness prediction than most trial and error common approaches [35]. Authors in [36] comment that an efficient methodology is needed to obtain optimal values for various parameters in artificial neural networks. In their paper, authors used the Gray–Taguchi method to determine the optimal value of various ANN model input parameters trained by different algorithms in a 2.5D milling process modeling.

The results showed that the combination with RNA presents a better performance. In the search for better prediction values, some studies compare statistical methodologies to ANNs. Authors in [37] studied the AISI316L steel dry turning surface roughness prediction, by comparing ANNs to multiple regression methods. The results found by the methods indicate a better artificial neural networks accuracy. A study involving the prediction of surface roughness in red brass turning was developed using a comparison between a complete factorial planning and ANN. The finding was that, based on ANN percentage error and the regression model, the artificial neural network was considered more accurate than the regression model [38]. Authors in [39] developed a model to investigate C23000 turning cutting parameter effects. Artificial neural networks and multiple regression models were used to model cutting forces based on cutting parameters, using variance analysis. It was concluded that the ANN model was more accurate than the regression model. Another study compared the mathematical regression with ANN, in order to predict the wear of the AISI 304 cutting tool. Experimental design includes four factors at five levels. According to the authors, the artificial neural network model was capable of better tool wear predictions [40]. A tool wear prediction by artificial neural networks in the Aluminum 7075 milling was proposed by [41]. Authors subdivided the research using the Taguchi model and ANNs. It was found that ANN prediction correlates very well with experimental results. In a turning paper, DOE was employed to select factor levels that benefit network prediction skills, concluding that the DOE methodology constitutes a better approach to the design of RBF networks for roughness prediction than most trial and error approaches. However, a frequently cited ANN disadvantage is the lack of a systematic way to design high-performance networks [35]. Another study deals with AISI 1040 steel machining. Response surface and ANN approaches were used. According to the authors, both approaches predict surface roughness accurately [42]. The authors in [43] reviewed several publications dealing with modeling and roughness by ANNs in machining processes. The review showed that most of the paper had a roughness (R_a_) average prediction, and that little attention was given to the efficiency of the training. Researchers point out the definition of the ideal network topology as the main problem in roughness modeling. Optimization efforts are detected in a small number of publications, and comparisons between topology definition approaches are hardly found. Moreover, with regard to validation, most publications neglect or make this ability unclear. The use of the third validation data set can be found in only a few studies. The use of statistical evaluation to compare trained networks can be found in only one fifth of the peer-reviewed papers, in addition to the lack of a statistical evaluation comparing ANN-based models and models obtained by other methods. According to authors, the ANNs models´ accuracy are points that require more attention and many papers are presented only in graphic forms, thus lacking information as to the results’ reproduction. There is no standard procedure for choosing more appropriate ANN settings, thus becoming a difficult task that depends on many variables. The trial and error method is the procedure most commonly used to identify the best settings [44]. Based on the peer-reviewed literature, some areas of improvement are suggested, such as the no need to transform or change data, that reveal non-regular periodic movement. In addition, papers focus on network characteristics in a specification phase, with tests being performed to determine errors and R oscillations, and to validate a performance optimization model [45].

The objective of this paper is to evaluate the application of different MQL strategies in Ti-6Al-4V alloy machining, and to study whether the prediction of results between the ANNs approaches and factorial design describe similar values. The classical DOE approach and its comparison with a high number of different neural network architectures have not yet been studied. The number of comparative analysis studies between the DOE and ANN approaches is still limited. This paper includes training and testing of 15,000 artificial neural networks to minimize a systematic trial and error in the top milling of a Ti-6Al-4V alloy. An MQL valve was built for this purpose.

## 2. Materials and Methods

**Figure 1:**
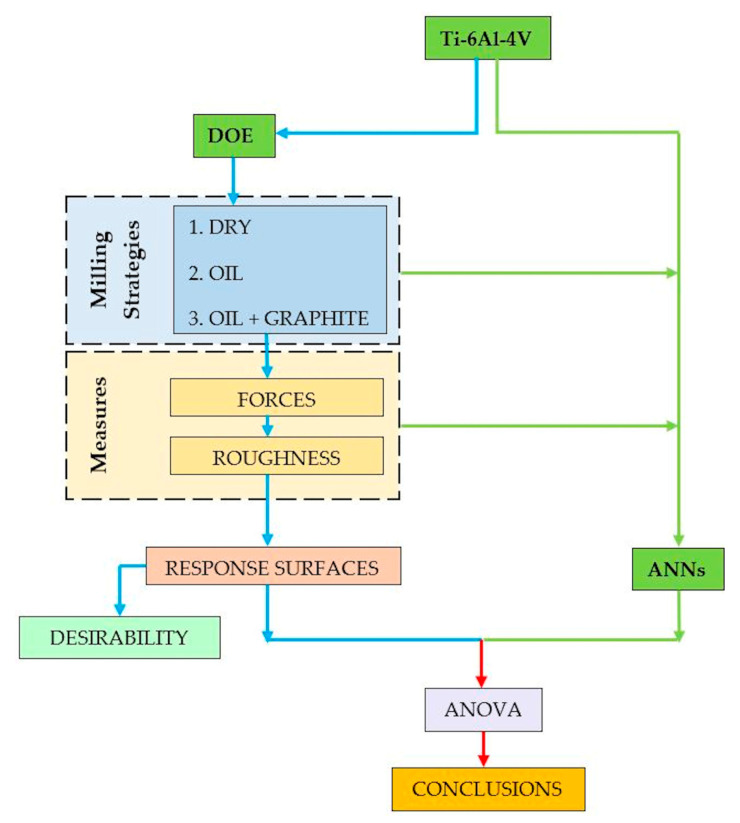
Experiment overview.

This was divided into two analyses: the first uses a factorial planning and its statistical correlations, and the second analysis uses artificial neural networks. Milling tests were performed using three different strategies. As results of the experimental planning, the influence of the independent variables on force and roughness parameters was verified by Pareto graphs. Statistical predictions were made using the generated response surfaces. The measured cutting and roughness data served to feed the artificial neural networks, and the predictions obtained by the two approaches were compared by means of a variance analysis. The desirability optimization function was used to seek optimal results aiming at lower roughness and lower cutting efforts. Figure 1 shows the experiment overview.

**Figure 2:**
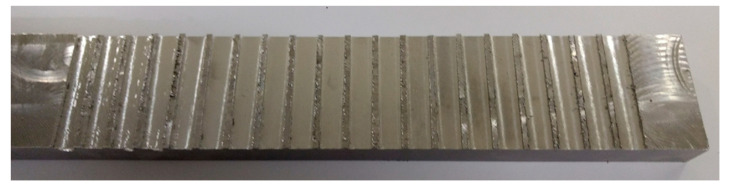
Ti-6AL-4V bar after machining.

The strategies and materials utilized to manufacture the MQL valve, milling trials, and training of the ANNs are described in this section. A commercial Ti-6Al-4V (Grade 5) titanium alloy bar of 500 mm × 50 mm × 17 mm with an average hardness of 32.4 Hardness Rockwell C was used in this study. Figure 2 illustrates the material with the machining tests performed. Table 1 shows the respective chemical composition.

### 2.1. Experimental Design and Machining

An experimental design of three factors and two levels (2^3^) with two central points was used, as shown in Table 2. A replication was performed for each cutting condition, totaling 60 tests. From these, the respective averages were calculated. The data adopted for the cutting speed (v_c_), feed rate (f), and depth of cut (a_p_) were recommended by the insert manufacturer. The procedure for using each cutting insert was used up to a wear of 0.1 mm.

The speed, feed rate, and depth of cut were considered as independent variables. As dependent variables, cutting force (F_c_), feed force (F_f_), penetration force (F_p_), torque (M_z_), root mean square height of the surface (S_q_), Skewness of height distribution (S_sk_) and Kurtosis of height distribution (S_ku_) were considered. Response surfaces were generated, and the desirability function was used to optimize the results of each strategy employed. Three different conditions were stipulated for the trials: (a) using no lubrication, called Strategy 1, (b) applying the designed mixing valve and adopting the MQL approach with oil (Strategy 2), and (c) using the MQL approach with the addition of 30% commercial graphite (Strategy 3). The trials were carried out according to Table 3. For the MQL approach, the Superfluid 3 lubricating oil from Quimatic/Tapmatic was applied. Its characteristics are listed in Table 4. The fluid was driven by a Magneti Marelli MAM00103 electric automotive pump (Mauá, Brazil), with a working pressure of 3 bar and a flow rate of 110 L/h connected to a 20 L fuel tank, and with an oil return circuit to the tank. The flow regulation was performed by a manual control valve set to 110 mL/h. The compressed air pressure was maintained at 6 bar.

**Figure 3:**
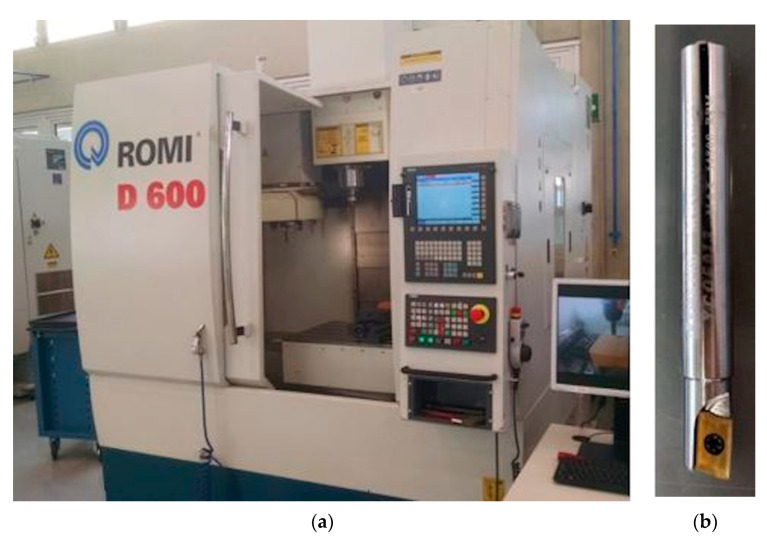
(**a**) ROMI D600 machining center and (**b**) KYOCERA tool used for milling.

The ROMI D600 machining center (Santa Bárbara d’Oeste, Brazil) with the SIEMENS 840-D CNC control (Figure 3a), a 20 hp motor, and total installed power of 30 kVA was used for milling tests. The insert BDMT 11 03 02 ER-JS (CVD) with grade CA6535 from KYOCERA (Sorocaba, Brazil) was chosen for milling (Figure 3b).

**Figure 4:**
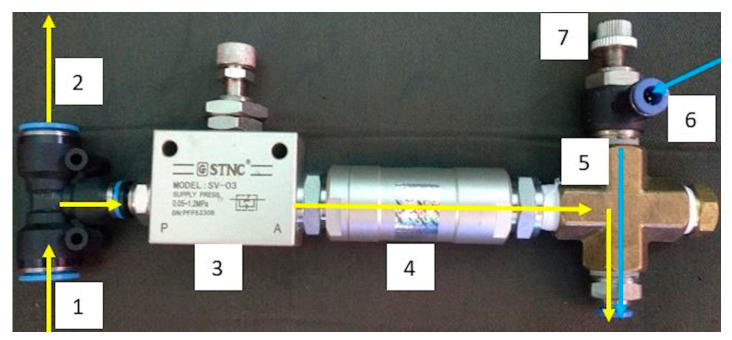
MQL valve circuit. (1) oil inlet; (2) return hose; (3) regulating valve; (4) check valve; (5) hydraulic equal cross coupling—air + oil mixture; (6) inlet compressed air; (7) control valve for compressed air.

The MQL valve circuit (Sao Caetano do Sul, Brazil) is illustrated in Figure 4. The needle is connected to the bottom part of hydraulic equal cross coupling through a hose, and directs the flow of the mixture.

**Figure 5:**
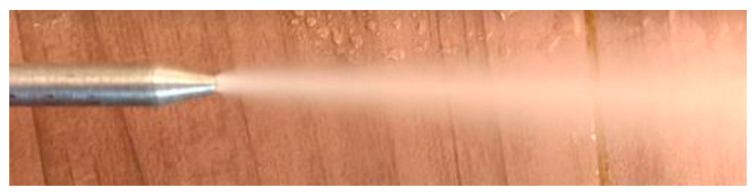
Needle and jet of Superfluido 3.

**Figure 6:**
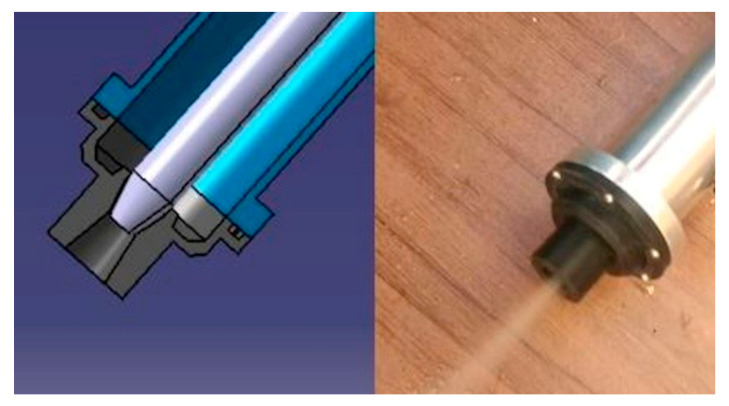
CAD design and the assembled valve.

The needle and jet of fluid can be seen in Figure 5. The CAD design and the assembled valve can be seen in Figure 6. The valve was attached to a lubrication hose that was adequate to the machining center.

### 2.2. Measurements

**Figure 7:**
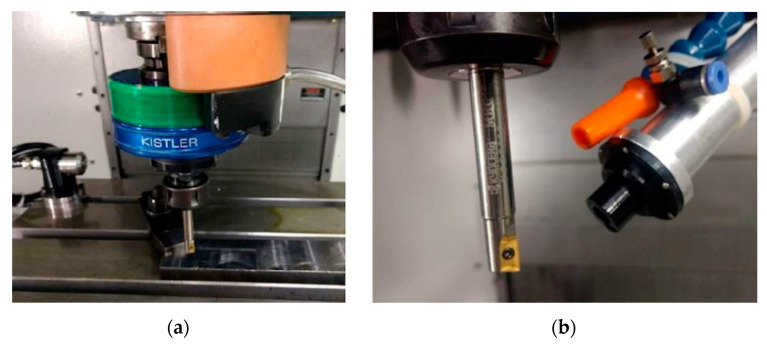
(**a**) The KISTLER rotary dynamometer. (**b**) the tool and the mounted valve.

**Figure 8:**
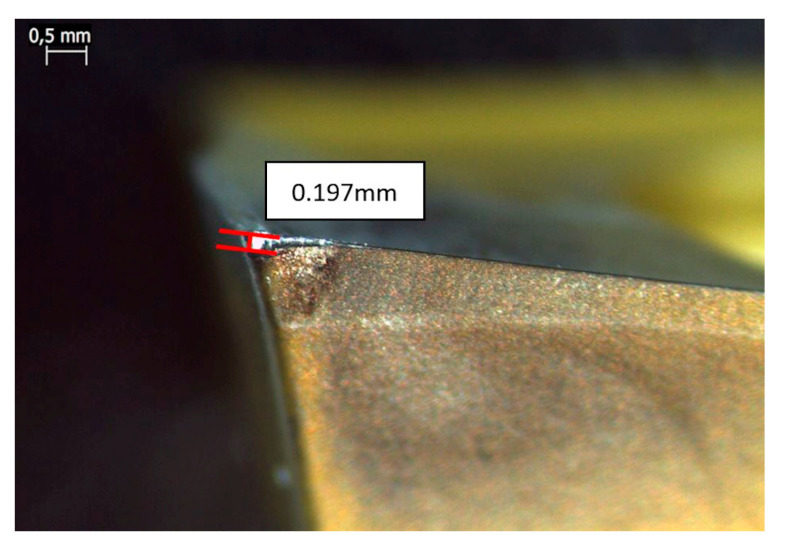
Wear measurement example.

For the measurement of forces during machining, a KISTLER 9123C rotary dynamometer (Sao Caetano do Sul, Brazil) with 5223B signal conditioner (Sao Paulo, Brazil) was used. Figure 7a shows the dynamometer mounted on the machine, and Figure 7b shows the tool and the mounted valve. The acquisition of the signals was carried out by the PCI 4472 card (Sao Caetano do Sul, Brazil), together with the Labview Signal Express 2.5 software from National Instruments. The frequency for data acquisition was 100 kHz. A Leica S6D microscope (Sao Paulo, Brazil) was used to check the wear after each machining, guaranteeing a V_b_ flank wear of up to 0.2 mm. Figure 8 shows the measurement of an insert.

**Figure 9:**
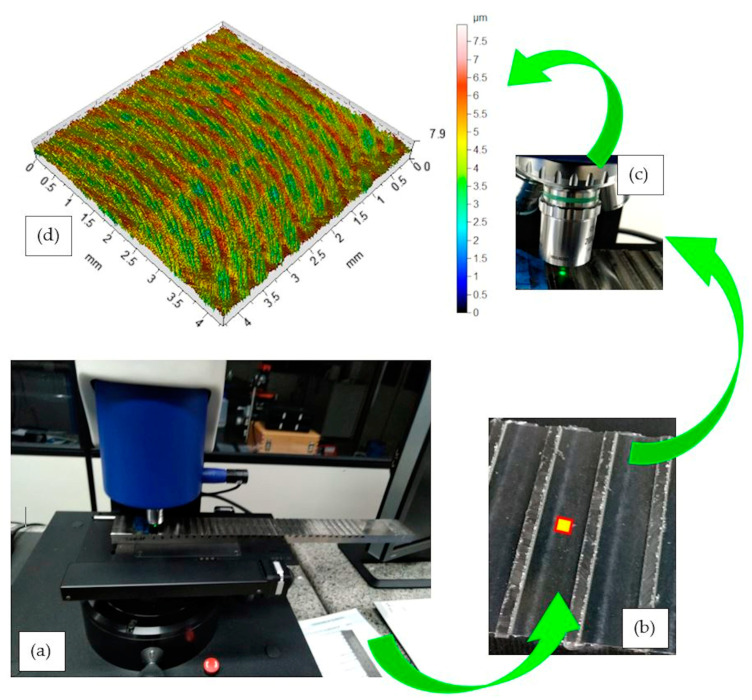
Roughness measurement procedure. (**a**) Taylor Hobson/Talysurf CCI Lite white light interferometer (**b**) Position and application area. (**c**) Measurement being performed. (**d**) Milled 3D surface topography.

The Talysurf CCI Lite white light interferometer (Sao Paulo, Brazil) was used for surface measurement. The surface roughness parameters generated using the software TalyMap Gold v. 6.2 and considered in this work were: S_q_ (root mean square height of the surface), S_sk_ (skewness of height distribution) and S_ku_ (kurtosis of height distribution). The measuring area was 4.3mm^2^ with used 0.8mm cut-off. Figure 9 illustrates the measurement procedure.

### 2.3. Artificial Neural Networks

From the machining data obtained by the experimental design and measurements, artificial neural networks were created for each cutting condition. With the “Automated Neural Network” workbench from Statistica software (version 13.5), multilayer perceptron networks (MLP) in three independent variables as input were created, and with the machining forces and roughness measured as the “target.” Table 5 indicates the independent and dependent variables’ selection of the networks used.

The MLP architecture has a feedforward characteristic, with a one-way sequence between layers. Neurons have activation functions that use input signals of the anterior layer in a mathematical function, and the choice of activation function influences the behavior of the neural network. The most commonly used ANN type is feedforward, with input, hidden, and output layers. Network data are entered through the input layer and each neuron in this layer relates to each input variable. Hidden layers transmit input data to output layers by accurately simulating the function of the original system. The output layer is related to the results of the network, based on the predicted results errors and the weights of the connections values between neurons. The function of each neuron contains synapses, sums function, and transfer function. Through connections, neurons communicate, multiplying the input value with weight coefficients. These signals are transformed by an activation function, generating the network output result [46]. For each machining strategy, 5000 networks were trained, using various combinations of neuron numbers, activation functions and number of layers, until they reached satisfactory values. Table 6 shows the values used for the training, testing, and validation data for each chosen strategy.

**Figure 10:**
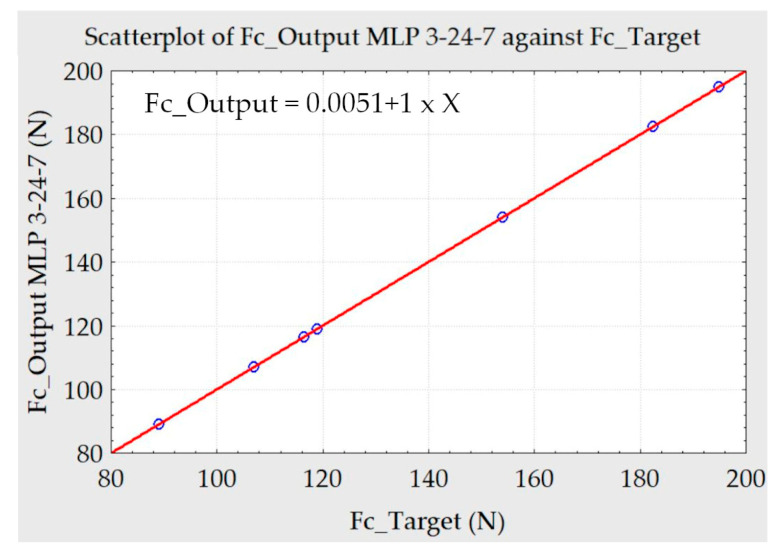
Scatterplot of Fc_Target x Fc_Output ANN.

Various training and tests were carried out using the test averages to validate the networks’ efficiency. Among the averages, three samples were randomly chosen for the tests, and seven samples were used for training. They were replicated 15 times, totaling 112 samples for training. The networks chosen were the ones that obtained the best results in training and testing, as exemplified in Figure 10.

The minimum and maximum number of neurons in the hidden layer were 3 and 25, respectively. The error function chosen was the sum of squares (SOS), due to the fact that it is one of the most used in training of artificial neural networks in regression tasks. The weight decay factor was stipulated between 0.0001 and 0.001. All available activation functions (identity, logistic, tanh, exponential and sine) were used for training.

### 2.4. Comparative Analysis

With the tabulated data, statistical tests were performed to verify the homogeneity of variances and to compare the pairs of averages. At the end of any experimental design, it should be checked whether the mathematical model obtained is valid [47]. Statistical tests were used to determine whether the two prediction strategies combined with the measurement produce equivalent estimates, i.e., to determine if there is a difference between the averages of results of the artificial neural networks and the experimental design. Analysis of variance was used, and therefore it can be seen if there are statistical differences between the predictions.

In addition, the desirability optimization function was used, in order to obtain optimal values for the three cutting conditions. The approach used in the desirability optimization consists of converting each response of the experimental design y_i_ into a function d_i_, varying between 0 and 1. If the answer is the desired one, a value of d_i_ = 1 is accepted, otherwise, d_i_ = 0 [47]. In this way, the independent variables are chosen to maximize the global desirability function. In this work, d_i_ = 1 was chosen as the lowest value for machining forces and roughness.

## 3. Results and Discussion

This topic provides the experimental design used, and the results of the average measurements of the machining forces and roughness and of the influence of the machining parameters, in relation to the dependent variables. The architectures of the ANNs are demonstrated, and a comparison of the treatments used is made.

### 3.1. Statistical Results of Surface Roughness Measurements

**Figure 11:**
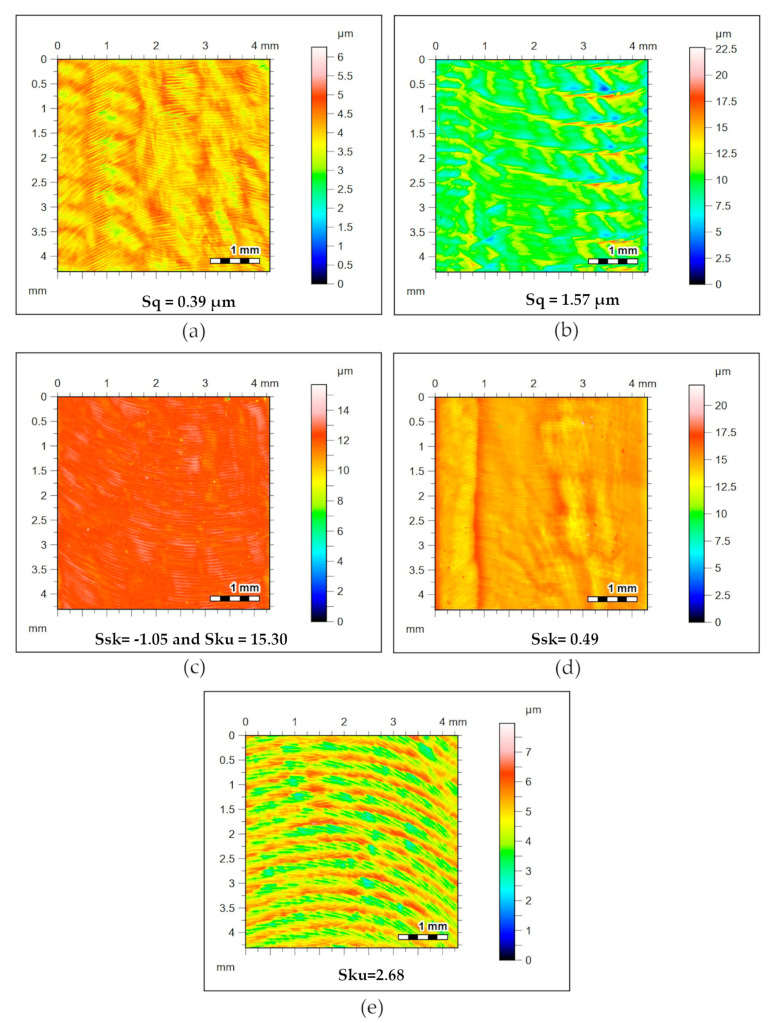
Sq, Ssk, Sku minimum and maximum milled surface topography parameter values measured. (**a**) S_q_ = 0.39 µm; (**b**) S_q_ = 1.57 µm; (**c**) S_sk_ = −1.05 and S_ku_ = 15.30; (**d**) S_sk_ = 0.49 µm; (**e**) S_sk_ = 2.68 µm

For every cutting condition, the maximum and minimum conditions of the parameters used were analyzed. The S_q_ parameter corresponds to the standard deviation of the distribution curve [48]. The S_ku_ parameter is always presented with the S_sk_ parameter, describing the shape of a topographic surface and its roughness distribution. Mathematically, the parameters skewness and kurtosis measure the symmetry and histogram deviation of all peaks and valleys heights of a machined surface in relation to the Gaussian distribution. The skewness roughness parameter can monitor machined surfaces’ wear and tear in service. A surface with a Gaussian distribution, which is symmetrically distributed, has the S_sk_ parameter equal to zero. Positive S_sk_ values indicate the predominance of high peaks, and negative values indicate the prevalence of valleys. On the other hand, S_ku_ parameter measures the degree of flattening or thinning of a topographic distribution of a peak´s roughness profile. On a surface with normal symmetric distribution, the S_ku_ parameter is equal to three. In practical terms, S_ku_ > 3 indicates the presence of acute peaks, while S_ku_ < 3 indicates surface texture free of disproportionately sharp peaks [49,50]. In this work, the lowest S_q_ value was obtained by the number 2 assay used in strategy 3 (0.39 µm). The maximum condition occurred in test 10 strategy 1 (1.57 µm). For the S_sk_ parameter, the minimum value occurred in test 1 strategy 3 (−1.05), and the maximum condition occurred in test 1 strategy 2 (0.49). As for the S_ku_ parameter, the minimum and maximum results are respectively found in test 2 strategy 1 (2.68) and test 1 strategy 3 (15.30). The statistical results are shown in Table 7. Based on statistical analysis results, the minimum and maximum topography surface values measured for each S_q_, S_sk_, S_ku_ parameter are shown in Figure 11.

### 3.2. Results and Analysis of Factorial Design

The use of experimental design resulted in measurements of the dependent variables for the three lubrication conditions. The data can be viewed in Table 8. The analysis of the feed rate, cutting, and penetration machining forces for the three tests, as illustrated in the following items, showed that these forces are dominated by the feed rate and the penetration depth variables. It is known that the greater these parameters are, the greater the chip removal forces are and, consequently, the greater the forces generated to remove them are. It has been noted that the cutting force decreases strongly in applications with oil and oil + graphite in relation to the cut without using fluid, due to the fluid emulsivity, minimizing friction, and temperature, acting directly on the flank of the cutting insert. The type of base fluid has a strong effect on the components of the cutting force. Consequently, the force is significantly reduced [1]. For the roughness parameter, the main factors that influence it are the feed rate, the cutting speed, the depth of cut, the vibrations of the cutting tools, the lubricant conditions, and the tool specifications [19]. In this study, it was found that the feed rate and a_p_ were the variables that most influenced roughness.

#### 3.2.1. Trial without Lubrication

**Figure 12:**
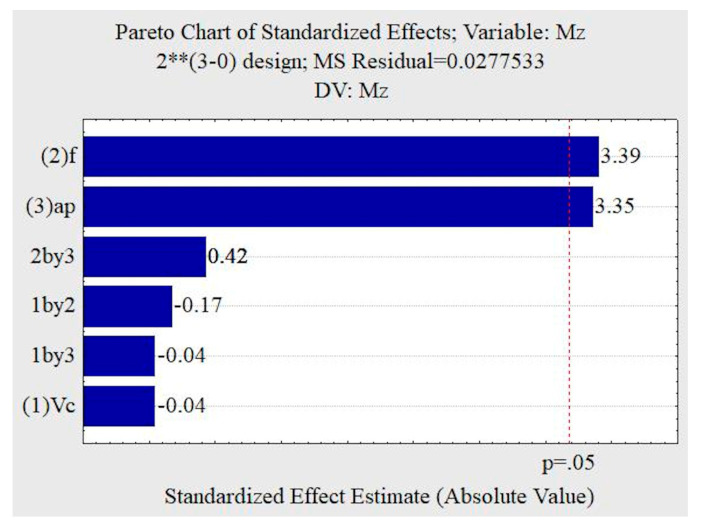
Pareto chart for the dry machining condition: torque M_z_.

Based on the Pareto graphs, it was observed that, for a 95% confidence interval, the cutting speed, advancing and forces were not influenced by the dependent variables. The roughness parameters also did not show any influence on the confidence interval adopted, although the ap and f are the variables with the greatest influence. Analyzing the illustration in Figure 12, the torque was influenced by the feed rate f and the depth of cut a_p_.

**Figure 13:**
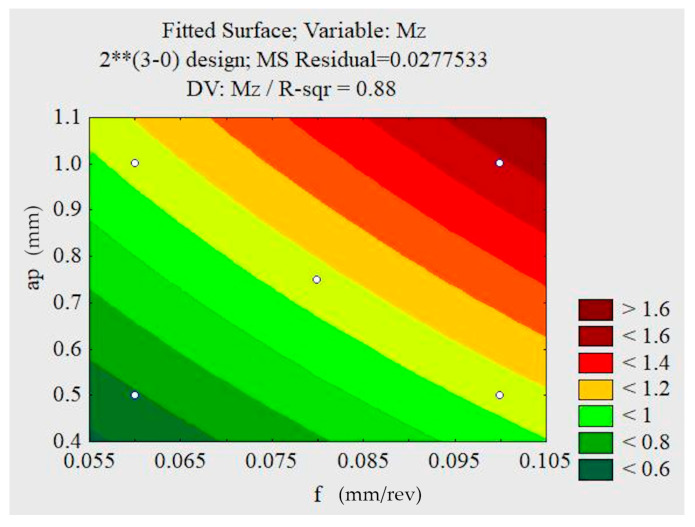
Response surfaces for the non-lubricated condition (M_z_).

In analyzing the response surfaces obtained by the factorial design of the test without lubrication illustrated in Figure 13, it can be seen that the minimum torque values occur with lower feeds, and lower depths of cut. The analysis process discards the statistical value of v_c_, since it does not influence the torque in the studied confidence interval. The lowest values of cutting forces, feed rate force, penetration force, and torque occur in smaller feeds, smaller depths of cut, and lower cutting speeds. The contributions of a_p_ and f are striking, indicating that they are possibly responsible for the mechanism regulating the machining forces of this test condition. The area of the section of cut that is defined by the product of the feed by the depth of cut increases with the increase of the feed and the depth of cut, causing an increase in the forces in question.

#### 3.2.2. Trial with Oil

**Figure 14:**
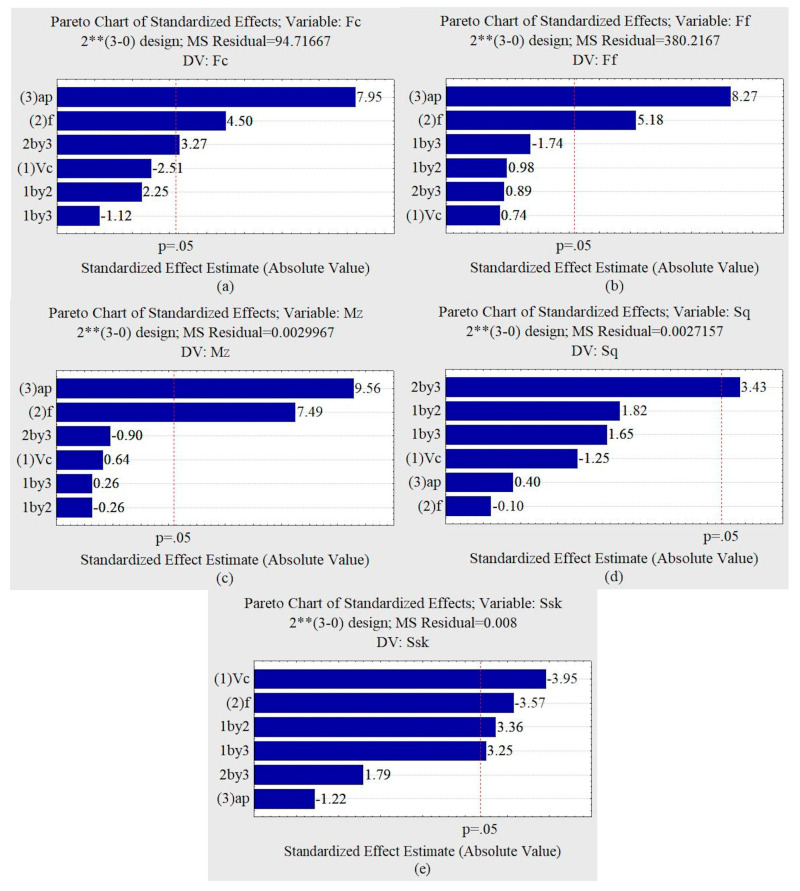
Pareto charts for oil machining condition. (**a**) Force F_c_. (**b**) Force F_f_. (**c**) Torque M_z_. (**d**) Roughness S_q_. (**e**) Skewness of height distribution.

Based on the Pareto graphs illustrated in Figure 14, it was observed that the variable a_p_, the advance f, and the interaction a_p_ and f demonstrated an influence on the cutting force. The variables a_p_ and f demonstrated an influence on the advance force for the 95% confidence interval.

For the same confidence interval, the penetration force was not influenced by the independent variables. Advancement was the most influential variable when a lower confidence interval was adopted.

The torque was influenced by the depth of cut and the feed. For the studied interval, the roughness S_q_ was influenced by interaction ap and f. The S_sk_ parameter was influenced by the cutting speed, the feed rate and the interactions between v_c_ and f and v_c_ and a_p_.

Analyzing the response surfaces obtained by the test´s factorial with lubrication, it can be seen that the minimum S_q_ values also occur for lower f and higher a_p_.

**Figure 15:**
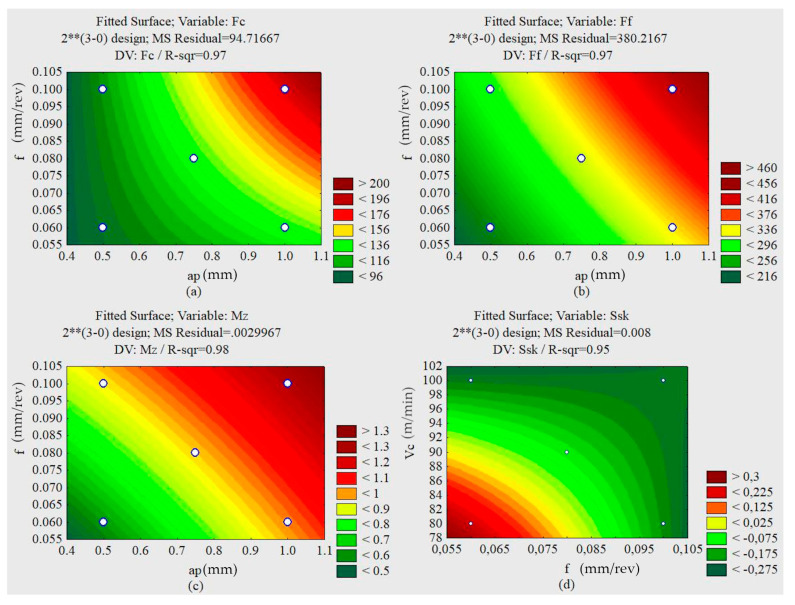
Response surfaces for the oil lubricated condition. (**a**) Cutting force F_c_ (a_p_ × f). (**b**) Feed force F_f_ (a_p_ × f). (**c**) Torque M_z_ (a_p_ × f). (**d**) Skewness (f × v_c_).

The lowest values of cutting forces and torque, illustrated in Figure 15, occur with smaller feed rates and smaller depths of penetration—discarding the cutting speed since it does not influence the approached parameter. The smallest feed rate forces, penetration forces, and torque occur with smaller depths of cut and smaller feed rates.

#### 3.2.3. Trial with Graphite

**Figure 16:**
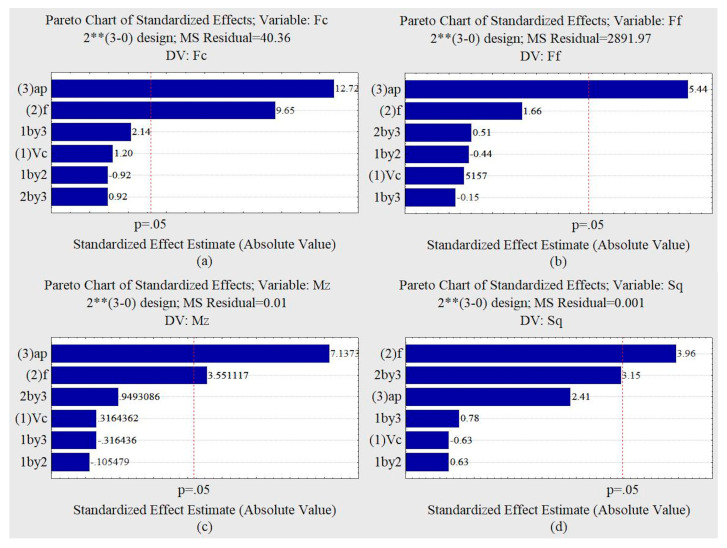
Pareto graphs for machining condition with graphite. (**a**) Cutting force F_c_. (**b**) Feed force F_f_. (**c**) Torque M_z_. (**d**) Roughness S_q_.

Based on the Pareto graphs illustrated in Figure 16, it was observed that the variable a_p_ and the feed rate showed an influence on the cutting force. The a_p_ variable showed an influence on the feed rate force, and for a 95% confidence interval, the penetration force was not influenced by the independent variables. The depth of cut was the variable of greatest influence for the penetration force. The torque was influenced by the depth of penetration and the feed rate. For the confidence interval adopted, the S_q_ roughness was influenced by the feed rate.

**Figure 17:**
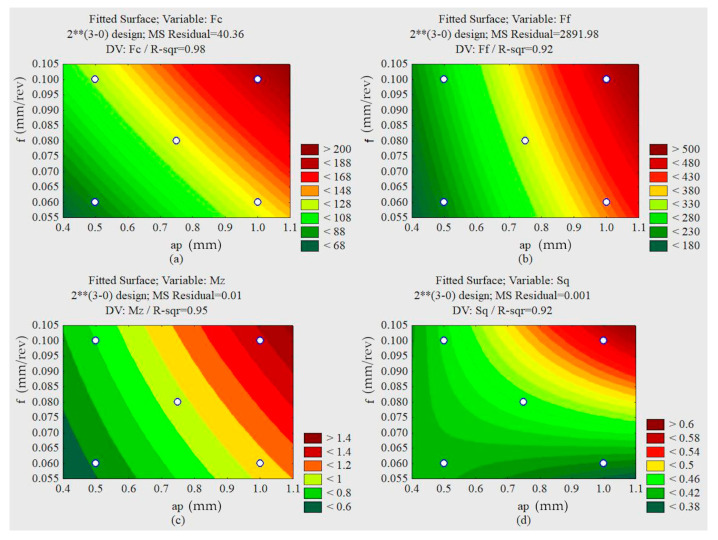
Response surfaces for the lubrication condition with graphite. (**a**) Cutting force F_c_ (a_p_ × f). (**b**) Feed force F_f_ (a_p_ × f). (**c**) Torque M_z_ (a_p_ × f). (**d**) Roughness S_q_ (a_p_ × f).

Analyzing the response surfaces obtained by the factorial design of the trial with oil and graphite illustrated in Figure 17, it can be seen that the minimum roughness values occur with lower feed rates and smaller depths of cut. The lower values of the cutting forces, feed rate forces, penetration forces, and torque occur with smaller feed rates, smaller depths of penetration, and lower cutting speeds.

Table 9 lists the functions of the responses of the variables F_c_, F_f_, F_p_, M_z_, S_sk_, S_ku_ and S_q_, obtained by the application of the factorial plan in this work.

In tests with no lubrication, it can be perceived that the machining forces (F_c_, F_f_, F_p_) are not statistically influenced by the independent variables. Only torque was influenced by the parameters f and a_p_ in the confidence interval studied. In the tests with lubrication, both with oil and graphite, it is apparent that machining forces and torque are significantly influenced by the parameters a_p_ and f. The S_q_ roughness was influenced by the interaction between a_p_ and f in the oil assay, while in the graphite assay, only the f parameter showed significant influence.

The roughness increased with higher feed rates. With the cut depth and feed increase, the cutting area in the tool is increased and, with this, the cutting effort is increased. In the oil strategy, the minimum Ssk values occur with higher cutting speeds and greater feed rates. This is due to a reduction in plastic deformation in the cutting zone incurred by a higher cutting speed. The surface defect is smaller, with a lower overall roughness value. Surface roughness decreases with increased cutting speed due to lower plastic deformation in the cutting zone associated with high speeds [51]. With the increase of the cutting speed, the temperature is increased, and the force is minimized by heating the material, helping its removal, and reducing roughness that, in turn, depends on feed rates. Oil application contributes to this process, minimizes temperature and favors tool life. Tool wear and coolant use generally influence surface finish [52]. This can be seen with the lowest roughness values in the strategies using oil and graphite-added oil. Lower cutting forces and lower feed rates provided lower roughness values. The analysis infers that the cutting force corroborates the surface roughness analysis [52].

#### 3.2.4. Machined Surface Roughness Analysis

**Figure 18:**
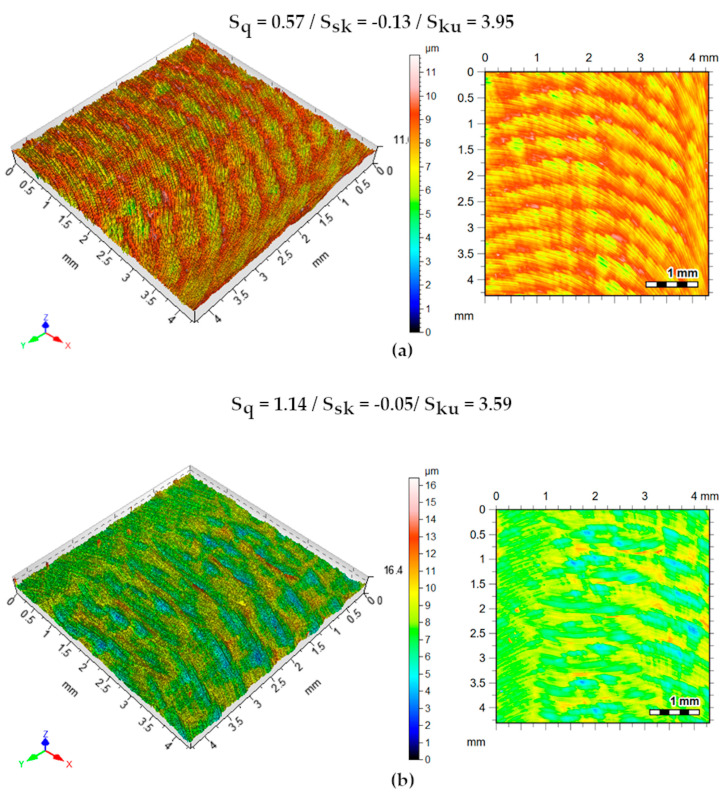
Machined surface topography. Isometric views and contour maps—dry strategy. (**a**)v_c_ = 80 m/min, a_p_ = 0.5 mm, f = 0.06 mm/rev.; (**b**) v_c_ = 100 m/min, a_p_ = 1.0 mm, f = 0.1 mm/rev.

Roughness parameters were shown to be governed by the independent variables a_p_ and f. The variable v_c_ is only noted in this work influencing S_sk_ in Strategy 2. To verify the roughness parameters behavior studied, the study of the lowest and highest values of the cut-off conditions was adopted as a premise. Thus, tests 1 and 8 of each experimental planning strategy are analyzed. Figure 18 shows the isometric surfaces and contour maps of the tests for the dry strategy. An increase of S_q_ in 99.8% was perceived due to the increase in a_p_ and f causing greater irregularities of the roughness profile. The negative S_sk_ parameter for both conditions indicates the prevalence of valleys. The positive S_ku_ parameter indicates that the peaks of the machined surface are sharp.

**Figure 19:**
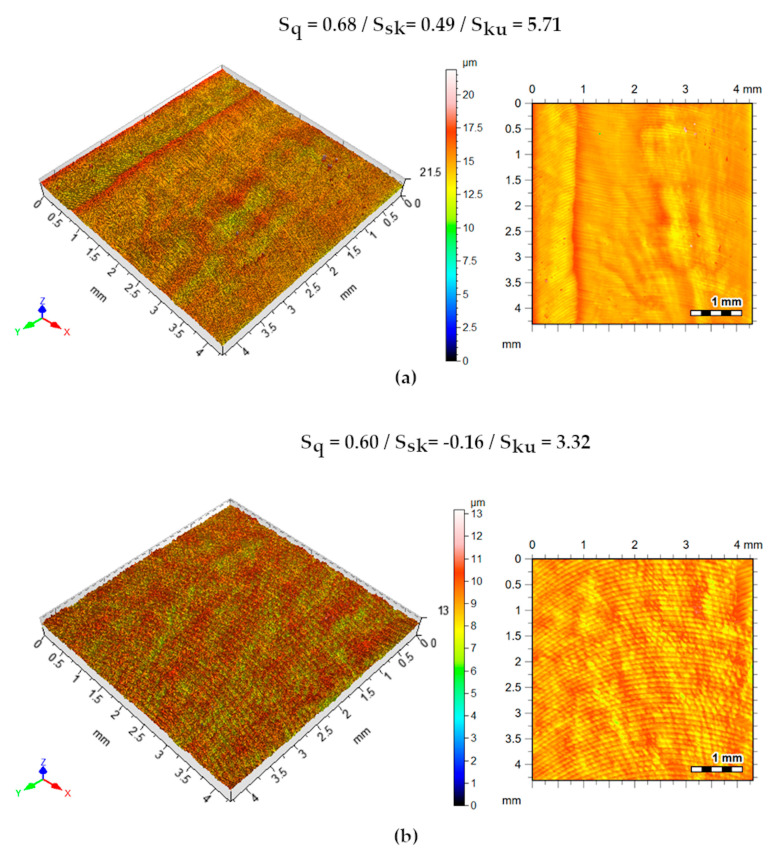
Machined surface topography. Isometric views and contour maps—oil strategy. (**a**)v_c_ = 80 m/min, a_p_ = 0.5 mm, f = 0.06 mm/rev.; (**b**) v_c_ = 100 m/min, a_p_ = 1.0 mm, f = 0.1 mm/rev

Figure 19 shows the isometric surfaces and contour maps of superfluid tests. The S_sk_ parameter, positive for the minimum cut-off condition, indicates the prevalence of peaks, which does not occur for the maximum values. The positive S_ku_ parameter indicates that the peaks of the machined surface are sharp. It is observed that the increase in feed and cutting speed accentuated the milling marks.

**Figure 20:**
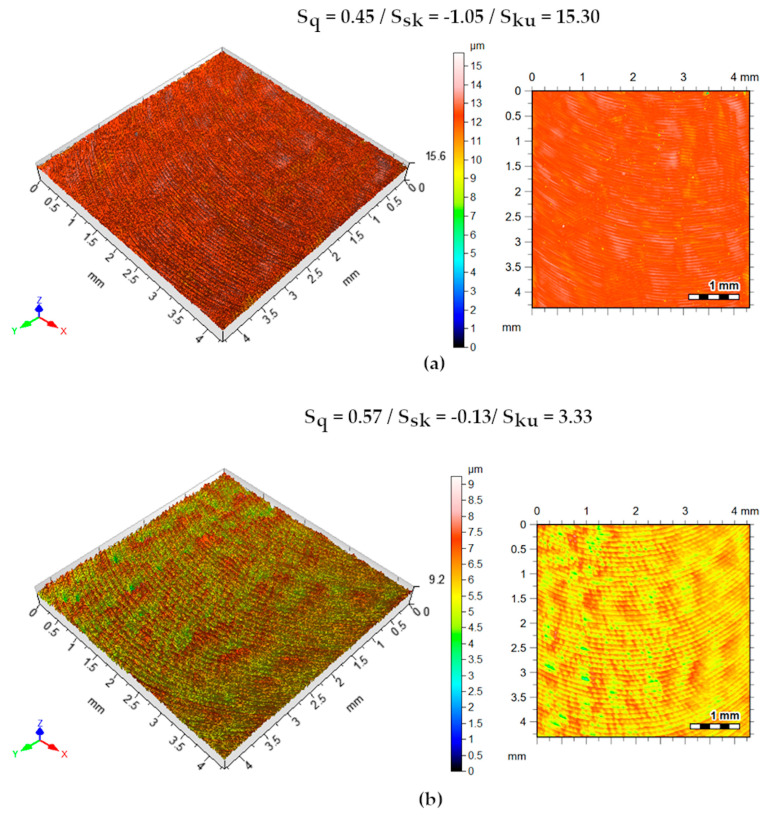
Machined surface topography. Isometric views and contour maps—oil + graphite strategy. (**a**) v_c_ = 80 m/min, a_p_ = 0.5 mm, f = 0.06 mm/rev.; (**b**) v_c_ = 100 m/min, a_p_ = 1.0 mm, f = 0.1 mm/rev.

Figure 20 shows the isometric surfaces and contour maps of the tests with the mixture of Superfluid 3 with graphite. An increase of S_q_ of 27% is noted. The S_sk_ parameter, negative for both cutting conditions, indicates the prevalence of valleys. The positive S_ku_ parameter indicates that the peaks of the machined surface are sharp. It is observed that the increase of f accentuated the advance marks.

#### 3.2.5. Desirability Function

**Figure 21:**
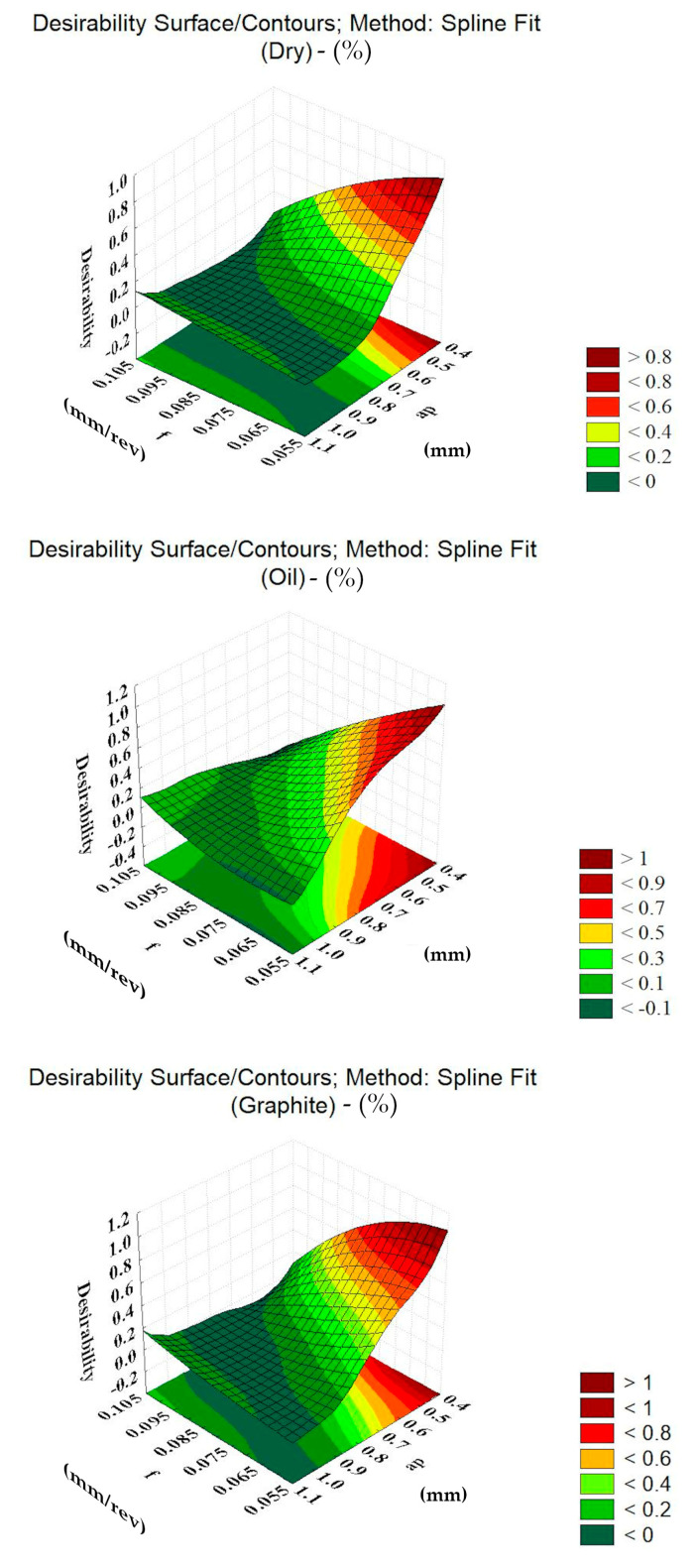
Desirability response surfaces.

Once you have the answer functions for all variables dependent on the research, an optimization bringing together all the variables allows you to show the best values for the application of the independent variables. The desirability function is used in single and multiple objective functions. Unlike other strategies, this optimization function avoids a conflict of responses. The variation of the input parameters must be planned to allow for working within the specified experimental design interval [17]. Using the Statistica software, optimal values were sought using the desirability function for each dependent variable. Table 10 and Figure 21 illustrate the results.

### 3.3. Prediction of Results by Artificial Neural Networks

With the data obtained by measuring the machining forces and roughness, different training standards and trials of the artificial neural networks were performed. Trainings were performed for different lubrication conditions with an average of 10 samples, creating supervised networks with three input variables (v_c_, f, and a_p_). As the target, the dependent samples F_f_, F_c_, F_p_, M_z_, and R_a_ were selected.

For the execution of the training and tests, the Automated Neural Network tool of the Statistica software was used. The tests represent the result of the simulation of the previously trained networks. For a future test to validate the training, three samples were randomly reserved. The remaining 7 samples were replicated 15 times, totaling 112 samples for training. Table 11 shows the variables chosen randomly for the validation tests. Table 12 contains information on the architectures of the artificial neural networks used to predict.

The test results can be seen in Table 13 and Table 14. The measured values and the results of the ANNs are listed respectively as output. There is a good approximation between the values.

### 3.4. Comparative Analysis of Predictions

**Figure 22:**
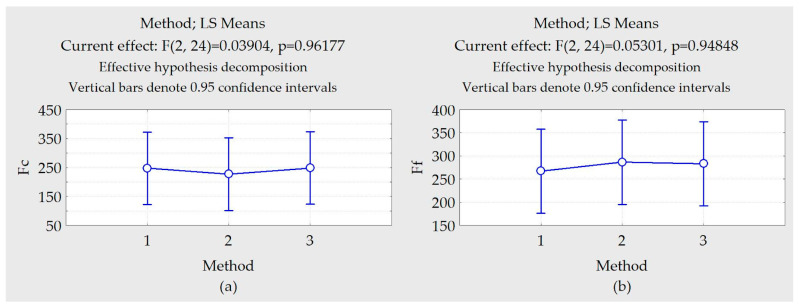

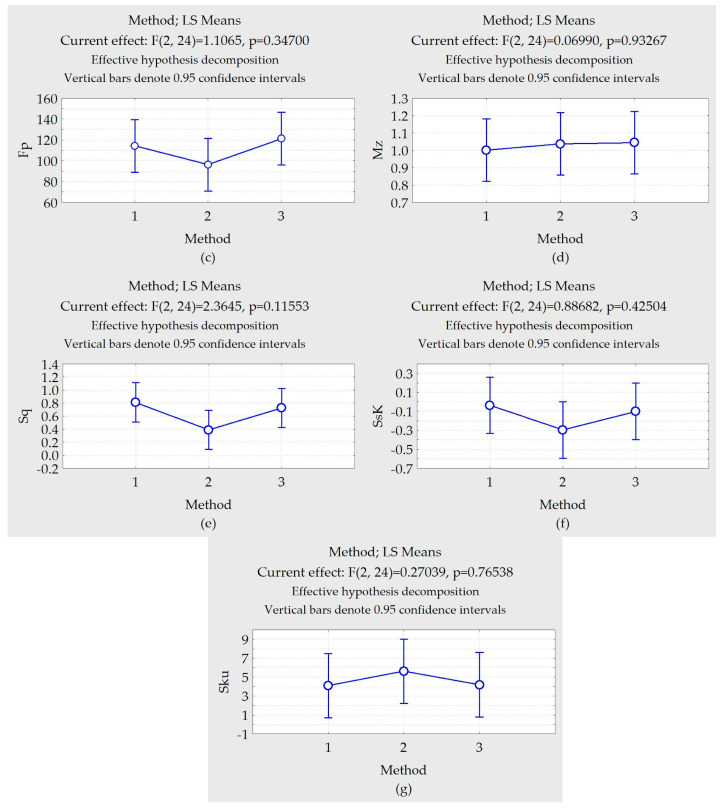
Differences between pairs of averages. (**a**) Cutting force F_c_ (**b**) Feed force F_f_. (**c**) Penetration Force F_p_. (**d**) Torque M_z_. (**e**) Roughness S_q_. (**f**) Skewness. (**g**) Kurtosis.

To verify whether the prediction strategies used in this work produce similar responses, a statistical test was carried out to verify the equality of treatment averages (ANOVA). It was found, as shown in Figure 22, that there were no global differences between prediction strategies (*p*-value > 0.05). In these graphs, the numbers 1, 2, and 3 represent the measured values, the results of the experimental design using the equations, and the results of the ANNs, respectively.

Future research can use statistical tests such as Duncan, Bonferroni, Tukey, and the validation in the case of differences in averages that they indicate, can be verified by the Levene test. The inclusion of other percentages of graphite and its comparison with other lubrication strategies, such as liquid nitrogen under dripping, would be interesting.

## 4. Conclusions

The design of the MQL mixing valve made it possible to study and analyze different lubrication conditions. The assembled circuit was based on commercial parts. It was found that the independent variables that most influenced the cutting forces and roughness were the depth of cut and feed rate. The cutting forces were significantly reduced with the use of the MQL approach, probably allowing for heat reduction in the cutting flank of the insert, due to the fluid emulsivity. The lowest average of the S_q_ roughness was achieved by using the MQL practice with cutting fluid and graphite. The desirability function provided the optimized search for the lowest cutting forces and for a lower roughness value, aiming at the surface finish. In tests with no lubrication, it can be perceived that the machining forces (F_c_, F_f_, F_p_) are not statistically influenced by the independent variables. Only torque was influenced by the parameters f and a_p_ in the confidence interval studied. In the tests with lubrication, both with oil and graphite, it is apparent that machining forces and torque are significantly influenced by the parameters a_p_ and f. The S_q_ roughness was influenced by the interaction between a_p_ and f in the oil assay, while in the graphite assay, only the f parameter showed significant influence. Lower cutting forces and lower feed rates provided lower roughness values. The paper took into account surface finishing cutting conditions. In the lowest feed rate and depth cut, it can be perceived that statistically, for the confidence interval adopted, that the forces had lower statistical influences than variables a_p_ and f. It has been observed that variables a_p_ and f define the cutting geometry, ergo being responsible for the roughness. The S_sk_ values showed values close to zero in most tests, indicating a symmetry between peaks and valleys. Negative values, however, indicate a trend of valley predominance and a concentration of material near the surface. All S_ku_ parameters measured demonstrated positive values, showing that the tests presented centralized acute peaks. With sharp peaks, there is the possibility of premature wear and tear in contact with another surface. The statistical comparison between the experimental design adopted and the artificial neural networks provided similar responses.

## Figures and Tables

**Table 1:** Chemical composition of Ti-6Al-4V (provided by VINER Brasil Tecnologia Ltda., Sao Paulo, Brazil).

Composition	Ti	Al	V	Fe	H	N	O	C	Y
**Data (%)**	Balance	6.49–6.56	4.03–4.14	0.16–0.19	0.002–0.003	0.003–0.004	0.192–0.196	0.024–0.028	<0.001

**Table 2:** Factorial design used for machining.

Tests	v_c_ (m/min)	f (mm/rev)	a_p_ (mm)
1	80	0.06	0.5
2	100	0.06	0.5
3	80	0.1	0.5
4	100	0.1	0.5
5	80	0.06	1.0
6	100	0.06	1.0
7	80	0.1	1.0
8	100	0.1	1.0
9	90	0.08	0.75
10	90	0.08	0.75

**Table 3:** Lubrication strategies used (dry/oil/oil + graphite).

Strategy	Cooling and Lubrication Condition
1	Dry (only compressed air)
2	Oil (minimum quantity lubrication (MQL))
3	Oil + Graphite (MQL)

**Table 4:** Characteristics of the cutting fluid used (provided by QUIMATIC/TAPMATIC).

Parameters	Properties
Aspect	Clear green viscous liquid
Scent	Characteristic, light
Density at 25 °C (g/mL)	0.88–0.91
Corrosion test on iron chips	No corrosion after 2 h
Viscosity centistokes, 25 °C	20–35
Acidity index	10–20

**Table 5:** Select variables for analysis.

Train, Test and Validation—ANNs	Variables
Continuous inputs	v_c_, f, a_p_
Continuous targets	F_c_, F_f_, F_p_, M_z_, S_q_, S_sk_, S_ku_

**Table 6:** Sampling Method (Random).

Train sample size (%)	70
Test sample size (%)	20
Validation sample size (%)	10

**Table 7:** Descriptive statistics—roughness.

Variable	Mean	Minimum	Maximum	Std. Dev.
S_q_ (µm)	0.69	0.39	1.57	0.37
S_sk_	−0.14	−1.05	0.49	0.26
S_ku_	4.35	2.68	15.30	2.22

**Table 8:** Results of factorial design used.

**Dry**	**v_c_ (m/min)**	**f (mm/rev)**	**a_p_ (mm)**	**F_c_ (N)**	**F_f_ (N)**	**F_p_ (N)**	**M_z_ (Nm)**	**S_q_ (µm)**	**S_sk_**	**S_ku_**
1	80	0.06	0.5	258.97	71.00	52.04	0.62	0.57	−0.13	3.95
2	100	0.06	0.5	228.50	108.00	60.96	0.68	0.80	−0.17	2.68
3	80	0.1	0.5	364.12	112.99	93.74	1.03	0.82	−0.20	3.06
4	100	0.1	0.5	427.50	109.00	113.53	0.97	1.01	−0.29	3.34
5	80	0.06	1.0	371.80	94.73	63.35	1.01	1.13	−0.06	3.18
6	100	0.06	1.0	396.81	100.50	70.97	0.98	1.31	0.09	3.65
7	80	0.1	1.0	510.50	172.62	119.37	1.44	1.46	0.04	3.07
8	100	0.1	1.0	531.50	139.40	103.14	1.45	1.14	−0.05	3.60
9	90	0.08	0.75	570.50	164.42	203.95	1.14	1.51	−0.09	4.30
10	90	0.08	0.75	617.50	207.94	207.94	1.31	1.57	0.36	5.39
**Oil**	**v_c_ (m/min)**	**f (mm/rev)**	**a_p_ (mm)**	**F_c_ (N)**	**F_f_ (N)**	**F_p_ (N)**	**M_z_ (Nm)**	**S_q_ (µm)**	**S_sk_**	**S_ku_**
1	80	0.06	0.5	117.00	216.50	84.80	0.59	0.68	0.49	5.71
2	100	0.06	0.5	89.00	260.00	95.20	0.64	0.46	−0.28	4.08
3	80	0.1	0.5	107.00	285.00	110.00	0.95	0.44	−0.17	3.62
4	100	0.1	0.5	116.00	310.00	113.00	0.93	0.45	−0.32	4.26
5	80	0.06	1.0	154.00	365.00	99.40	1.01	0.47	−0.01	4.37
6	100	0.06	1.0	116.50	315.00	96.00	1.03	0.46	−0.17	4.13
7	80	0.1	1.0	195.00	412.50	124.00	1.25	0.57	−0.24	3.54
8	100	0.1	1.0	182.50	435.00	129.00	1.30	0.60	−0.17	3.32
9	90	0.08	0.75	125.50	330.00	137.60	0.88	0.48	−0.19	4.61
10	90	0.08	0.75	119.00	335.00	125.00	0.91	0.46	−0.08	3.59
**Oil + Graphite**	**v_c_ (m/min)**	**f (mm/rev)**	**a_p_ (mm)**	**F_c_ (N)**	**F_f_ (N)**	**F_p_ (N)**	**M_z_ (Nm)**	**S_q_ (µm)**	**S_sk_**	**S_ku_**
1	80	0.06	0.5	80.00	192.50	52.00	0.55	0.45	−1.05	15.30
2	100	0.06	0.5	84.00	217.50	63.00	0.63	0.39	0.00	3.28
3	80	0.1	0.5	127.50	242.50	71.00	0.77	0.45	0.01	3.76
4	100	0.1	0.5	115.00	255.00	81.00	0.78	0.44	−0.27	4.11
5	80	0.06	1.0	127.50	375.00	77.00	1.04	0.41	−0.48	6.17
6	100	0.06	1.0	142.50	410.00	88.00	1.02	0.40	−0.23	4.06
7	80	0.1	1.0	175.00	485.00	112.00	1.34	0.56	−0.06	3.26
8	100	0.1	1.0	190.00	465.00	121.00	1.36	0.58	−0.13	3.33
9	90	0.08	0.75	130.00	410.00	124.00	1.08	0.44	−0.30	4.73
10	90	0.08	0.75	140.00	395.00	116.00	1.06	0.40	−0.34	5.11

**Table 9:** Statistical functions for the prediction of dependent variables—Mathematical Function.

**Dry**	**Mathematical** **Function**
F_c_	= 253.250 − 3.99675 × v_c_ − 867.125 × f + 56.15 × f × v_c_ + 0.655 × a_p_ × v_c_ 768.999 × a_p_ × f + 201.2475
F_f_	= −451.195 + 6.336 × v_c_ + 4113.75 × f − 49.990 × f × v_c_ − 3.023 × a_p_ × v_c_ + 1845.0 × a_p_ × f + 133.2
F_p_	= −212.050 + 2.299 × v_c_ + 1984.197 × f − 8.112 × f × v_c_ − 1.865 × a_p_ × v_c_ − 151.680 × a_p_ × f + 148.705
M_z_	= −0.435 + 0.0045 × v_c_ + 10.75 × f − 0.05 × f × v_c_ − 0.0009 × a_p_ × v_c_ + 5.0 × a_p_ × f + 0.36
S_q_	= −4.94 + 0.05 × v_c_ + 39.99 × f −0.34 × f × v_c_ − 0.028 × a_p_ × v_c_ − 7.4 × a_p_ × f + 3.01
S_sk_	= −0.59 + 0.006 × v_c_ + 12.475 × f − 0.185 × f × v_c_ + 0.009 × a_p_ × v_c_ + 3.7 × a_p_ × f − 0.595
S_ku_	= 17.67 − 0.15 × v_c_ − 93.925 × f + 1.00125 × v_c_ × f + 0.099 × a_p_ × v_c_ + 1.80 × a_p_ × f − 6.64
**Oil**	**Mathematical Function**
F_c_	= 375.025 − 2.8 × v_c_ − 4400 × f + 38.750 × f × v_c_ − 1.550 × a_p_ × v_c_ + 2250.0 × a_p_ × f + 51.75
F_f_	= −41.225 + 1.412 × v_c_ − 2168.750 × f + 33.750 × f × v_c_ − 4.800 × a_p_ × v_c_ + 1225.0 × a_p_ × f + 421.
F_p_	= 13.775 + 0.580 × v_c_ + 298.750 × f + 0.625 × f × v_c_ − 0.590 × a_p_ × v_c_ + 365.0 × a_p_ × f + 34.95
M_z_	= −0.554 + 0.0017 × v_c_ + 12.125 × f − 0.0250 × f × v_c_ + 0.002 × a_p_ × v_c_ − 3.5 × a_p_ × f + 0.63
S_q_	= 3.48 − 0.025 × v_c_ − 24.69 × f + 0.168 × f × v_c_ + 0.012 × a_p_ × v_c_ + 12.625 × a_p_ × f − 1.55
S_sk_	= 9.01 − 0.087 × v_c_ − 63.04 × f + 0.541 × f × v_c_ + 0.042 × a_p_ × v_c_ + 11.525 × a_p_ × f − 3.627
S_ku_	=20.85 − 0.15 × v_c_ − 155.44 × f + 1.43 × f × v_c_ + 0.03 × a_p_ × v_c_ + 6.60 × a_p_ × f − 3.06
**Oil + Graphite**	**Mathematical Function**
F_c_	= 14.96 − 0.350 × v_c_ + 1703.125 × f − 10.312 × f × v_c_ + 1.925 × a_p_ × v_c_ + 412.50 × a_p_ × f − 69.0
F_f_	= −414.31 + 4.875 × v_c_ + 3921.875 × f − 42.187 × f × v_c_ − 1.125 × a_p_ × v_c_ + 1937.5 × a_p_ × f + 270.0
F_p_	= −27.625 + 0.7 × v_c_+243.75 × f − 1.875 × f × v_c_ − 0.05 × a_p_ × v_c_ + 775.0× a_p_ × f + 6.0
M_z_	= −0.438 + 0.006 × v_c_ + 2.937 × f − 0.019 × f × v_c_ − 0.004 × a_p_ × v_c_ + 6.75 × a_p_ × f + 0.66
S_q_	= 1.191 − 0.006 × v_c_ − 6.425 × f + 0.036 × f × v_c_ + 0.0036 × a_p_ × v_c_ + 7.25 × a_p_ × f − 0.59475
S_sk_	= −12.026 + 0.117 × v_c_ + 106.056×f − 1.03 × f × v_c_ − 0.03 × a_p_ × v_c_ − 6.67 × a_p_ × f + 2.589
S_ku_	= 140.035 − 1.26 × v_c_ − 1039.62 × f + 9.081 × f × v_c_ + 0.48 × a_p_ × v_c_ + 176.87 × a_p_ × f − 46.74

**Table 10:** Results of the application of the desirability optimization function.

Trial	v_c_ (m/min)	f (mm/rev)	a_p_ (mm)	%Confidence
Dry	90.0	0.06	0.5	54.0
Oil	100.0	0.07	0.5	66.0
Oil + Graphite	100.0	0.06	0.5	69.0

**Table 11:** Samples chosen randomly for testing the training of the ANNs created.

**Dry**	**v_c_ (m/min)**	**f (mm/rev)**	**a_p_ (mm)**	**F_c_ (N)**	**F_f_ (N)**	**F_p_ (N)**	**M_z_ (Nm)**	**S_q_** **(µm)**	**S_sk_**	**S_ku_**
6	100	0.06	1.0	396.81	100.50	70.97	0.98	1.31	0.09	3.65
7	80	0.1	1.0	510.50	172.62	119.37	1.44	1.46	0.04	3.07
9	90	0.08	0.75	570.50	164.42	203.95	1.14	1.51	−0.09	4.30
**Oil**	**v_c_ (m/min)**	**f (mm/rev)**	**a_p_ (mm)**	**F_c_ (N)**	**F_f_ (N)**	**F_p_ (N)**	**M_z_ (Nm)**	**S_q_** **(µm)**	**S_sk_**	**S_ku_**
1	80	0.06	0.5	117.00	216.50	84.80	0.59	0.68	0.49	5.71
4	100	0.1	0.5	116.00	310.00	113.00	0.93	0.45	−0.32	4.26
9	90	0.08	0.75	125.50	330.00	137.60	0.88	0.48	−0.19	4.61
**Oil + Graphite**	**v_c_ (m/min)**	**f (mm/rev)**	**a_p_ (mm)**	**F_c_ (N)**	**F_f_ (N)**	**F_p_ (N)**	**M_z_ (Nm)**	**S_q_ (µm)**	**S_sk_**	**S_ku_**
2	100	0.06	0.5	84.00	217.50	63.00	0.63	0.40	0.00	3.28
7	80	0.1	1.0	175.00	485.00	112.00	1.34	0.56	−0.06	3.26
9	90	0.08	0.75	130.00	410.00	124.00	1.08	0.44	−0.30	4.73

**Table 12:** Summary of active networks.

Summary	Dry	Oil	Oil + Graphite
**Index**	4	2	1
**Network Name**	MLP 3-8-7	MLP 3-24-7	MLP 3-16-7
**Training**	1.0	1.0	1.0
**Test**	1.0	1.0	1.0
**Validation**	1.0	1.0	1.0
**Training error**	0.0	0.0	0.0
**Test error**	0.0	0.0	0.0
**Validation error**	0.0	0.0	0.0
**Training algorithm**	BFGS 237	BFGS 128	BFGS 68
**Error function**	SOS	SOS	SOS
**Hidden activation**	Tanh	Tanh	Exponential
**Output activation**	Identity	Identity	Identity

**Table 13:** Custom predictions—Forces and torque.

**Dry**	**F_c_**	**F_c__Output**	**F_f_**	**F_f__Output**	**F_p_**	**F_p__Output**	**M_z_**	**M_z__Output**
1	396.81	385.77	100.50	130.93	70.97	91.13	0.98	0.98
2	510.50	500.35	172.62	95.27	119.37	81.32	1.44	1.36
3	570.50	617.46	164.42	207.93	203.95	207.95	1.14	1.31
**Oil**	**F_c_**	**F_c__Output**	**F_f_**	**F_f__Output**	**F_p_**	**F_p__Output**	**M_z_**	**M_z__Output**
1	117.00	81.04	216.50	255.59	84.80	102.08	0.59	0.59
2	116.00	90.17	310.00	298.07	113.00	123.28	0.93	0.93
3	125.50	119.00	330.00	334.99	137.60	124.99	0.88	0.91
**Oil + Graphite**	**F_c_**	**F_c__Output**	**F_f_**	**F_f__Output**	**F_p_**	**F_p__Output**	**M_z_**	**M_z__Output**
1	84.00	102.16	217.50	296.71	63.00	90.20	0.63	0.75
2	175.00	199.59	485.00	533.07	112.00	154.14	1.34	1.51
3	130.00	139.99	410.00	394.99	124.00	115.99	1.08	1.06

**Table 14:** Custom predictions—Superficial Roughness.

**Dry**	**S_q_**	**S_q__Output**	**S_sk_**	**S_sk__Output**	**S_ku_**	**S_ku__Output**
1	1.31	1.46	0.09	−0.03	3.65	2.81
2	1.46	0.93	0.04	−0.11	3.07	3.98
3	1.51	1.57	−0.09	0.35	4.30	5.39
**Oil**	**S_q_**	**S_q__Output**	**S_sk_**	**S_sk__Output**	**S_ku_**	**S_ku__Output**
1	0.68	0.37	0.49	−0.04	5.71	4.17
2	0.45	0.46	−0.32	−0.12	4.26	3.33
3	0.48	0.46	−0.19	−0.07	4.61	3.58
**Oil + Graphite**	**S_q_**	**S_q__Output**	**S_sk_**	**S_sk__Output**	**S_ku_**	**S_ku__Output**
1	0.40	0.35	0.00	−0.55	3.28	7.20
2	0.56	0.52	−0.06	−0.01	3.26	2.14
3	0.44	0.41	−0.30	−0.33	4.73	5.10

## Nomenclature

ANN	Artificial Neural Networks
ANOVA	Analysis of variance
a_p_	Depth of cut (mm)
BPNN	Backpropagation neural network
CVD	Chemical vapor deposition
CNC	Computer numeric controlled
DOE	Design of experiments
f	Feed rate (mm/rev)
F_c_	Cutting force (N)
F_f_	Feed force (N)
F_p_	Penetration force (N)
GA	Genetic algorithm
MQL	Minimum quantity lubrication
M_z_	Torque (Nm)
MLP	Multi Layer Perceptron
S_ku_	Kurtosis of height distribution (dimensionless)
SOS	Sum of squares error function (dimensionless)
S_sk_	Skewness of height distribution (dimensionless)
S_q_	Root mean square height of the surface (µm)
v_c_	Cutting speed (m/min)
V_b_	Flank wear (mm)
2(**)	two levels
